# Trypanosome Mitochondrial Translation and Tetracycline: No Sweat about Tet

**DOI:** 10.1371/journal.ppat.1005492

**Published:** 2016-04-21

**Authors:** Hassan Hashimi, Sabine Kaltenbrunner, Alena Zíková, Julius Lukeš

**Affiliations:** 1 Institute of Parasitology, Biology Centre, Czech Academy of Sciences, University of South Bohemia, Czech Republic; 2 Faculty of Science, University of South Bohemia, Czech Republic; 3 Canadian Institute for Advanced Research, Toronto, Canada; University of Wisconsin Medical School, UNITED STATES

## Overview

A recent study vividly demonstrates the unintended impact of the antibiotic tetracycline (Tet) on animal and plant mitochondrial translation, which corresponds to the α-proteobacterial origin of the organelle. This effect was ultimately manifested by an impact on the cellular, and even organismal, levels in the studied eukaryotes. Thus, widespread use of Tet in agriculture and biomedical research is now under scrutiny. Interestingly, Tet does not affect this process in trypanosomatids. The highly divergent nature of trypanosomatid mitochondrial ribosomes may explain why these flagellates are insensitive to Tet.

## How Does Tetracycline Affect Mitochondria?

A study recently published by Moullan and coauthors [1] pronounced that even low doses in the μg/ml range of tetracycline (Tet) have an adverse effect on mitochondrial function in several model eukaryotes, ranging from metazoa to plants to in vitro human cultures. This paper brought into the limelight the danger of profuse usage of this class of antibiotics not only prophylactically, e.g., to maintain and promote growth in livestock, but also in biomedical research. The emergence of elegant platforms for Tet-controlled transcription by Tet-On and Tet-Off systems for inducing and suppressing gene expression, respectively, in a variety of eukaryotic models underlies the widespread use of this antibiotic in experimental biology. Importantly, as illustrated by these authors, even low, single-digit μg/ml concentrations of Tet also induced what has been termed “mitonuclear protein imbalance,” in which the proportion of nucleus-encoded proteins imported into the organelle versus those arising from mitochondrial genes increases [1]. This subtle but perceptible phenotype, long overlooked, consequently impairs mitochondrial functions, such as respiration, and also induces significant detrimental changes at the organismal level, such as diminished growth and delayed development. Interestingly, a beneficial impact was observed in *Caenorhabditis elegans*, in which treatment with the Tet-class antibiotic doxycycline (Dox) mitigated the age-related decline in motility. Thus, the authors concluded that the vast amount of data produced using Tet-controlled gene expression may be confounded by the unintended disruption of the given model’s mitochondria. They also cautioned against the future use of Tet-On and Tet-Off systems [1].

## How Does Tetracycline Inhibit Mitochondrial Translation?

**Fig 1:**
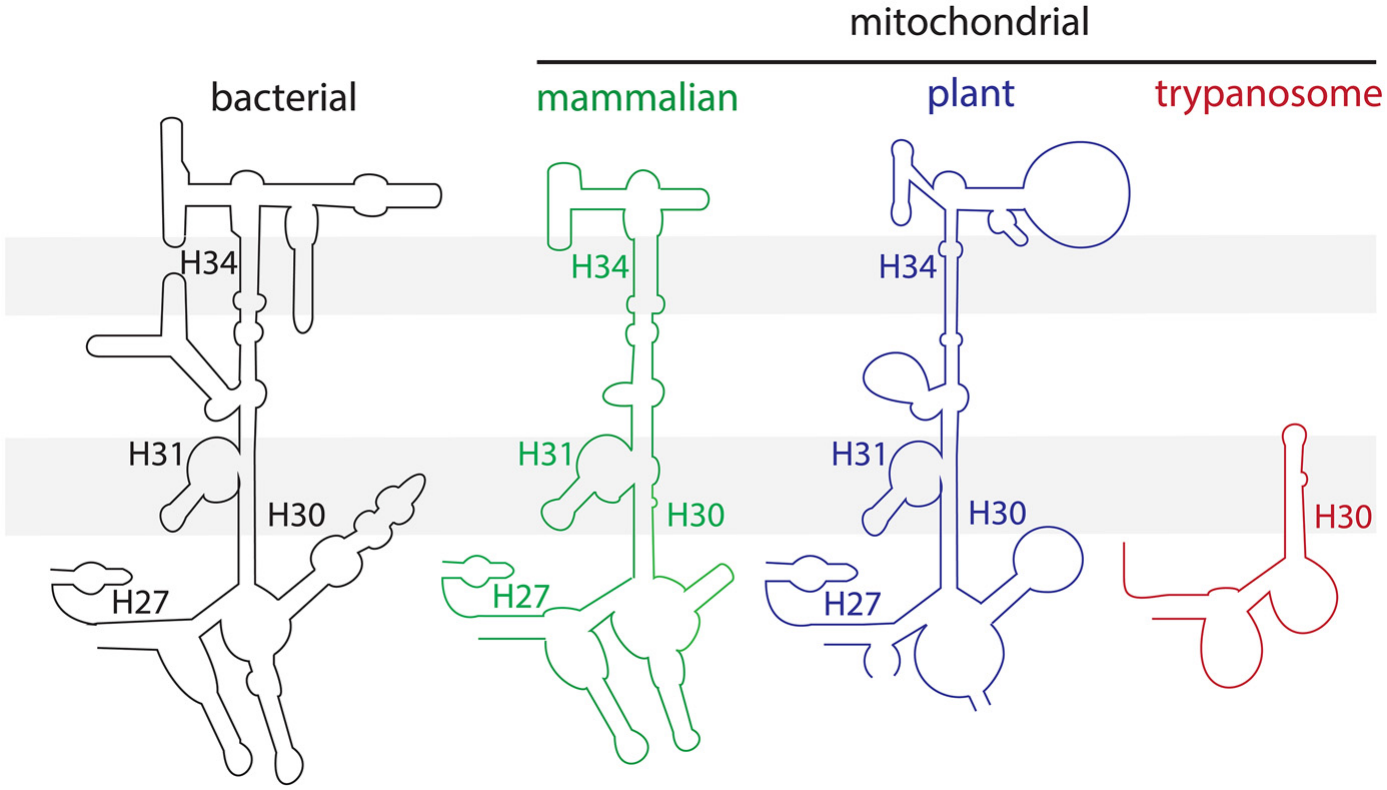
The ribosomal small subunit rRNA loops containing H31 and H34, as well as H27, double-stranded helices from bacteria (black) plus the mitochondria of mammals (green), plants (blue) and trypanosomes (red). Grey shading highlights the location of H31 and H34 in the SSU rRNAs bearing these motifs, as well as their absence in the same region of the trypanosomatid SSU rRNA. Helix H30, which is conserved throughout all the depicted rRNAs, is also indicated as a reference point. Adapted from [8] and [9].

The mitonuclear protein imbalance caused by the antibiotic in question arises from its long-ago established inhibition of mitochondrial translation [2], which coheres to the α-proteobacterial origin of the organelle. More specifically, Tet prevents the accommodation of aminoacylated (aa-) tRNA into its entry point to the mitochondrial ribosome, the A-site [3,4]. Two solved structures of the bacterium *Thermus thermophilis* 30S ribosomal small subunit (SSU) bound by Tet share two sites where the antibiotic attaches to facilitate its inhibitory action [5,6]. In the first location adjacent to the A-site, the compound intercalates into a pocket formed by the double-stranded (ds) helices H31 and H34 of 16S ribosomal (r) RNA, the polyribonucleotide component of the SSU, and binds to the sugar-phosphate backbone of H34. Within this position, Tet sterically hinders aa-tRNA attachment into the A-site of the ribosome, thus inhibiting translation [3,5,6]. A second Tet-binding position identified in both structures involves another ds 16S rRNA helix designated H27, a switch region that plays a role in selection of the proper aa-tRNA at the A-site [7]. Although this would not directly hinder aa-tRNA accommodation into the ribosome, it may still contribute to the disruption of this translational step [3]. These secondary structural motifs of the bacterial SSU 16S rRNA that interact with Tet are conserved in the homologous SSU rRNA of plant and animal mitochondrial ribosomes (Fig 1) [8,9]. Thus, the inhibitory effect of Tet on mitochondrial translation leading to the consequences described by Moullan and coauthors [1] could rely on a very similar mechanism as described for bacterial ribosomes [3,5,6].

## How Does Tetracycline Affect Trypanosomes?

**Fig 2:**
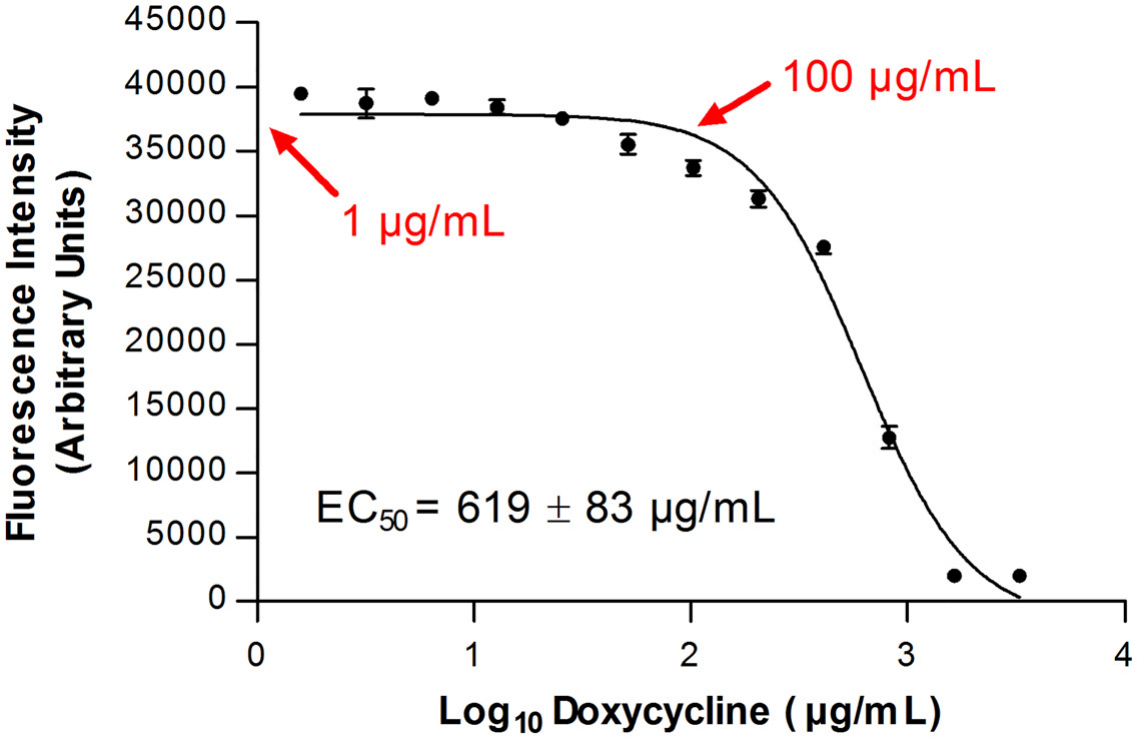
Effect of 24 hour doxycycline exposure on the viability of procyclic stage *T*. *brucei*. Data points represent the mean cell viability ± standard error of the mean (SEM) (*n* = 4), as measured by the Alamar Blue fluorescent dye assay. *X*-axis, μg/ml doxycycline (log scale); *y*-axis Alamar Blue fluorescence intensity in arbitrary units; doxycycline EC_50_ value calculated from curve given on lower left. Red arrows indicate points corresponding to 1 and 100 μg/ml concentrations on the *x*-axis. The assay was performed as previously described [19].

What does all this mean for the large community of molecular parasitologists studying *Trypanosoma brucei*? The development of Tet-controlled transcription for functional analysis of nuclear genes, mostly via straightforward application of RNA interference and the expression of exogenous genes, represented a major breakthrough for the field [10]. This platform has been so successful in *T*. *brucei* that it has also been implemented to study various *Leishmania* species [11]. However, are all these data, acquired over two decades, confounded by the recently reported Tet-triggered mitonuclear protein imbalance plaguing typical model systems of biomedical research [1]? Should the future application of this useful platform be reconsidered? Reassuringly, the answer to both questions is no. As seen in Fig 2, Dox exhibits a very high EC_50_ value of about 620 μg/ml in cultured procyclic *T*. *brucei*, the life cycle stage residing in the tsetse fly midgut that bears an actively respiring mitochondrion [12]. Indeed, up to 50 μg/ml of Dox does not negatively impact parasite fitness. This observation recapitulates tacit knowledge in the field that Tet treatment at the standard induction dose of 1 μg/ml, considerably lower than the aforementioned concentration, does not hamper *T*. *brucei* cell division. In contrast, when mitochondrial gene expression is down-regulated, ultimately decreasing the levels of the organellar gene products that are generated by mitochondrial ribosomes, an obvious growth-inhibition phenotype is observed (e.g., [13] and [14]).

## Is Trypanosome Mitochondrial Translation Affected by Tetracycline?

The seeming insensitivity of trypanosomatids to Tet treatment occurs because mitochondrial translation is not susceptible to the antibiotic. Studies done on procyclic *T*. *brucei* and the related species *Leishmania tarentolae* have demonstrated that their mitochondrial translation is not affected even when they are grown in the presence of 100 μg/ml Tet [13,15], a concentration greatly exceeding those used by Moullan and coauthors [1], but half that of the maximal concentration not affecting procyclic *T*. *brucei* fitness (Fig 2).

Could one of the mechanisms of bacterial Tet resistance, Tet efflux, Tet degradation, rRNA mutations, or the participation of ribosomal protection proteins (RPPs) [3,4] underlie the Tet resistance of trypanosomatid mitochondrial translation? Most RPPs are homologous to prokaryotic elongation factors EF-Tu and EF-G, a structural feature that allows these proteins to access and dislodge Tet from the ribosome A-site [3,4]. However, only genes encoding mitochondrial EF-Tu, EF-G1, and EF-G2 have been identified in trypanosomatid genomes [14], implying the lack of RPPs to perform the same function on trypanosomatid mitochondrial ribosomes. With the current state of knowledge, it is still not possible to rule out Tet efflux of the mitochondrion or Tet degradation within the organelle with confidence. However, available data allow exploring the last possibility that key differences in the SSU rRNA sequence may underlie the Tet insensitivity of trypanosomatid mitochondrial translation.

## Are Unique Features of Trypanosome Mitochondrial Ribosomes Responsible for the Insensitivity of Mitochondrial Translation to Tetracycline?

The mitochondrial ribosomes of both *T*. *brucei* and *L*. *tarentolae* are quite different from their counterparts in animals, plants, and bacteria. The 9S SSU and 12S large subunit (LSU) rRNAs are considerably reduced as compared to the rRNAs of aforementioned organisms, representing the smallest known orthologs of these molecules [9,16]. To compensate for this deficiency in the rRNA component of the ribosome, trypanosomatids have experienced an expansion in the number of mitochondrial ribosomal proteins, most of which are unique to these kinetoplastid flagellates. The solved structure of the *L*. *tarentolae* mitochondrial ribosome [9] further refines this information in terms of the lack of Tet-sensitivity of the ribosome. Here, we see that the loop of the 9S rRNA, which encompasses the important Tet-binding H31 and H34 helices present in other SSU rRNAs, is significantly truncated (Fig 1). This loop, which also contains rRNA elements normally needed for aa-tRNA accommodation into the A-site, is replaced in mitochondrial ribosomes by trypanosomatid-specific proteins [9]. In this milieu, the Tet-binding site is ablated by the lack of the H31 and H34 helices, the latter of which ordinarily provides the sugar-phosphate backbone for attachment of the antibiotic [3,5,6]. Furthermore, the H27 helix that represents another Tet-binding site is considerably reduced in trypanosomatid 9S rRNA.

The observation that the contact points for Tet-binding are lacking in the trypanosomatid mitochondrial SSU is not proof that these structural features are completely responsible for organellar translation’s insensitivity to treatment with this antibiotic. However, their conspicuous absence represents the most parsimonious hypothesis for this phenomenon considering the current state of knowledge. If this hypothesis is true, trypanosomatid mitochondrial ribosomes may be informative in comparative studies further investigating the mechanism of Tet inhibition of translation in other bacterial and organellar systems. Certainly, the insensitivity of trypanosomatid mitochondrial translation to Tet represents yet another exquisite example of the extreme evolutionary divergence of this group of protists, considering this trait is found in the bacterial domain of life, which gave rise to mitochondria, and remains conserved in the widely separated plant and mammalian eukaryotic clades. This phenomenon also belongs to a long line of discoveries made in trypanosomatids that have contributed to our understanding of biological processes vital to eukaryotes as a whole, epitomized by renowned examples, including the linkage of glycoproteins to the plasma membrane via glycosylphosphatidylinositol anchors [17] and the shaping of transcriptomes by RNA editing [18]. It is also reassuring to know that as we unravel more about the fascinating biology of trypanosomatids, our genetic tools are precise.
